# Sorafenib with concurrent multiple-line therapies improves overall survival in advanced stage hepatocellular carcinoma

**DOI:** 10.1097/MD.0000000000016074

**Published:** 2019-06-21

**Authors:** Pojen Hsiao, Kun-Chou Hsieh, Yaw-Sen Chen, Chia-Chang Hsu, Gin-Ho Lo, Yu-Chan Li, Pei-Min Hsieh, Hung-Yu Lin, Tsung-Chin Wu, Jen-Hao Yeh, Chih-Wen Lin

**Affiliations:** a Division of Gastroenterology and Hepatology, E-Da Dachang Hospital; b Department of Surgery, E-Da Hospital, I-Shou University; c School of Medicine, College of Medicine, I-Shou University, Kaohsiung; d Division of Gastroenterology and Hepatology, Department of Medicine; e Health Examination Center; f Graduate Institute of Medicine, College of Medicine, Kaohsiung Medical University, Kaohsiung; g School of Chinese Medicine, College of Chinese Medicine, China Medical University; h Research Center for Traditional Chinese Medicine, China Medical University Hospital, Taichung, Taiwan.

**Keywords:** advanced stage, hepatocellular carcinoma, immunotherapy, overall survival, sorafenib

## Abstract

The efficacy of sorafenib in combination with transarterial chemoembolization (TACE) or multiple-line therapies in patients with advanced hepatocellular carcinoma (HCC) remains unclear. This study aimed to investigate the overall survival (OS) of patients with advanced HCC in response to different combination therapies.

We analyzed the treatment and OS of 401 patients with Barcelona clinic liver cancer stage C HCC between 2012 and 2017. Mortality was analyzed using multivariate Cox regression, and OS was analyzed by the Kaplan–Meier method.

The mean age was 59 years and males were predominant. During a median follow-up time of 8.6 months (range, 1–80 months), 346 (86.2%) patients died. In the multivariate Cox regression analysis, primary tumor size ≥5 cm, serum alpha-fetoprotein ≥200, and serum albumin ≥3.5 were significantly associated with mortality. In addition, compared with sorafenib alone, multiple-line treatments with sorafenib and multiple-line treatments without sorafenib yielded significantly decreased mortality. In the Kaplan–Meier analysis, sorafenib with TACE, multiple-line treatments with sorafenib, third-line treatments with sorafenib, and multiple-line treatments without sorafenib yielded a significantly better median OS than sorafenib alone.

Sorafenib with concurrent multiple-line therapies significantly improved OS. These combination therapies will provide important information for immunotherapy combination with locoregional therapies in advanced HCC.

## 1 Introduction

Hepatocellular carcinoma (HCC) is the fifth most common cancer but the second leading cause of cancer-related death worldwide.^[1–3]^ HCC has a high correlation with viral- and alcohol-related cirrhosis^[4]^ and is the second leading cause of cancer-related death in Taiwan.^[5]^ Performing ultrasound and monitoring alpha-fetoprotein (AFP) in patients with an elevated risk of HCC is suggested,^[6–9]^ but only a small percentage of HCCs are diagnosed during routine monitoring. Therefore, a minority of HCC patients are considered for surgical resection.^[10,11]^ Additionally, most HCC patients are diagnosed at an intermediate or advanced stage, and one-third of patients are diagnosed with advanced stage HCC.^[12,13]^

Patients with macrovascular invasion (MVI) and/or extrahepatic spread (EHS) are classified as Barcelona clinic liver cancer (BCLC) stage C, and sorafenib is the only suggested therapy for BCLC stage C patients.^[7,14]^ Some studies have demonstrated that sorafenib increases overall survival (OS) in patients with MVI and/or EHS.^[8,15,16]^ However, the clinical prognosis and median OS of most patients after sorafenib treatment remain unsatisfactory. In attempts to improve the effects of sorafenib, several studies of subsequent or combination therapies with radiofrequency ablation (RFA),^[17]^ radiotherapy,^[18]^ hepatic artery infusion chemotherapy (HAIC),^[19]^ and systemic therapy^[20]^ have been performed. In addition, some reports have demonstrated that in BCLC stage C patients, the combination of sorafenib with transarterial chemoembolization (TACE) can improve OS compared with that obtained from sorafenib or TACE monotherapy.^[21,22]^ Recently, studies have shown that compared with administration of either sorafenib or TACE alone, the coadministration of conventional TACE and sorafenib increases the time to progression but does not increase OS in BCLC stage B/C patients.^[23–26]^ However, the effect of sorafenib in combination with TACE or other multiple-line therapies is still controversial and needs to be studied. Hence, this study aimed to identify clinical factors predicting OS. Moreover, we investigated the OS in patients with advanced HCC who underwent sorafenib monotherapy or sorafenib with concurrent multiple-line therapies.

## 2 Materials and methods

### 2.1 Patient enrollment

We retrospectively enrolled 401 BCLC stage C HCC patients with MVI or/and EHS between 2012 and 2017 at E-Da Hospital, I-Shou University, Kaohsiung, Taiwan. The study was approved by the Ethics Committee of E-Da Hospital (EMRP-107-121), and all of the participants supplied informed consent. Patients were diagnosed with HCC based on at least 1 typical HCC image or histological confirmation according to the recommendations of the AASLD.^[14]^ OS was defined the range from the time of inclusion to the time of the last follow-up or death, and follow-up was conducted until July 2018. Antiviral therapy was defined as nucleoside analogs administered to patients with hepatitis B virus (HBV) or Peg-interferon or direct-acting antiviral agents administered to patients with hepatitis C virus (HCV) according to the guidelines of the Taiwan Association for the Study of the Liver.

### 2.2 Treatment

Patients received 1 or more treatments, which were classified as sorafenib, surgical resection, RFA, TACE, radiotherapy, HAIC, and best supportive care (BSC). A total of 56.8% of patients initially received the standard dose (800 mg/d) of sorafenib, and 43.2% of patients initially received lower than standard doses. First-line, second-line, third-line, and fourth-line treatments were defined as therapies administered to patients who received 1 treatment, 2 treatments, 3 treatments, and 4 treatments, respectively. Multiple-line treatment was defined as the concurrent administration of 2 or more treatments. For sorafenib-based treatments, the treatments were categorized as sorafenib alone and as second-line, third-line, and fourth-line treatments with sorafenib.

### 2.3 Statistical analysis

Numerical data are expressed as the mean (ranges), and categorical data are described using numbers (percentages). Continuous variables were categorized based on the mean values or the limit of normal ranges in Cox regression models. Comparisons of continuous data were performed using the Pearson *χ*^2^ test, and categorical variables were analyzed using the Fisher exact test as appropriate. OS was calculated through a Kaplan–Meier analysis and was presented as the median and 95% confidence interval (CI). Cox proportional hazards regression analysis of OS in HCC patients was performed. A *P*-value of <.05 indicated statistical significance. All statistical analyses were conducted with Statistics Package for Social Science (SPSS) software (version 23.0, SPSS, Inc, Chicago, IL).

## 3 Results

### 3.1 Patient demographic data

The demographic data and clinical characteristics of 401 BCLC stage C patients are listed in Table 1. The mean age was 59 years, and males were predominant. In addition, 84.3% of the patients reported a history of HBV, 36.9% reported a history of HCV, and 52.9% reported alcohol use. Additionally, 16.7% of patients had received antiviral therapy. Approximately 73.1% of patients were Child-Pugh class A, and 75.6% had cirrhosis. Patients frequently exhibited large and multifocal HCCs. Patients with MVI and EHS accounted for 90.8% and 41.6% of the participants, respectively.

**Table 1:**
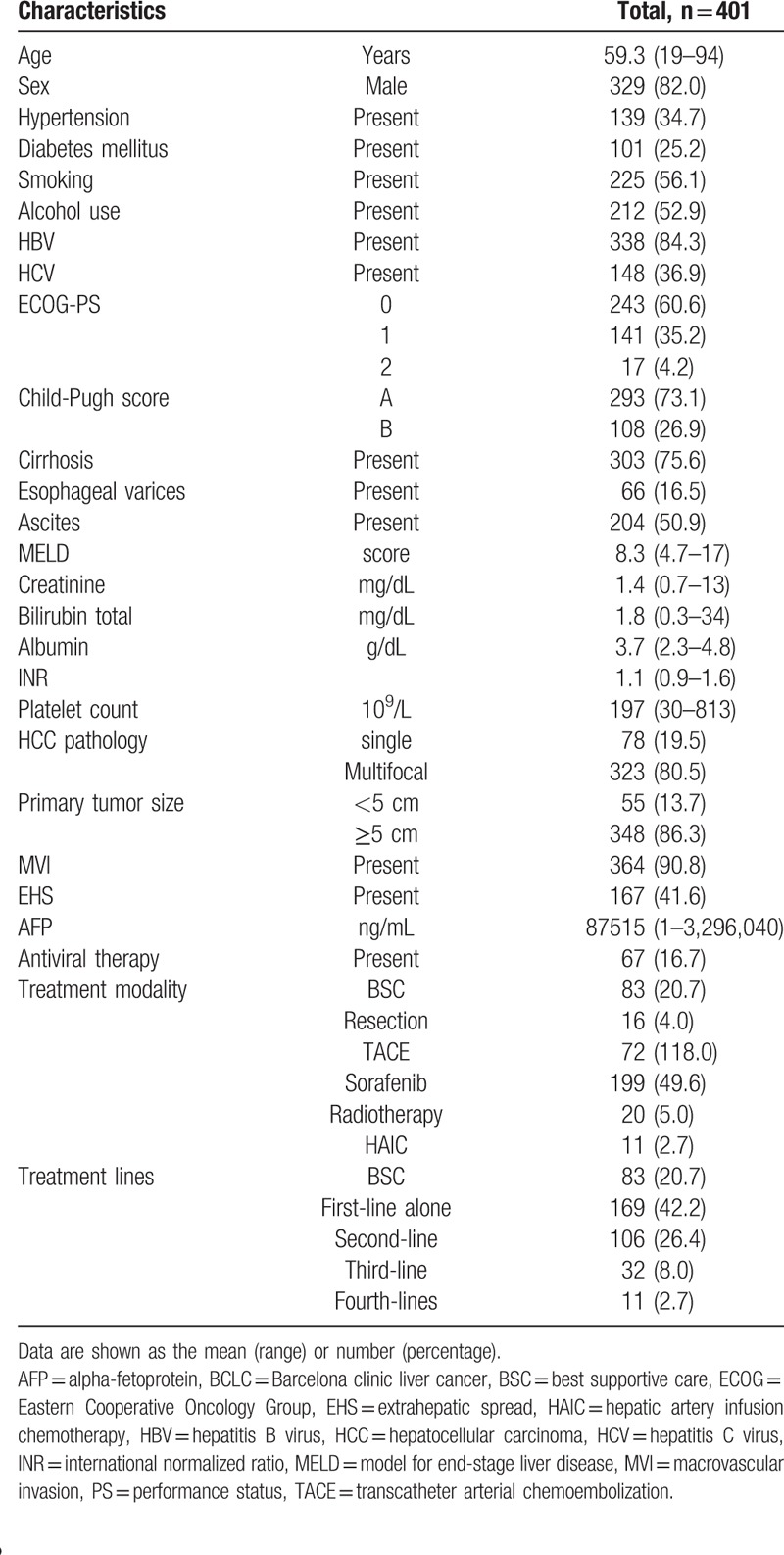
Demographic data and clinical features of 401 HCC patients.

### 3.2 Prognostic factors associated with mortality

During a median follow-up time of 8.6 months (range, 1–80 months), 346 (86.2%) patients died. The prognostic factors of mortality were analyzed in univariate analyses and are listed in Table 2. Mortality was significantly correlated with serum albumin, primary tumor size, serum AFP, treatment modality, treatment method and treatment line. In the multivariate Cox regression analysis, 3 models were used due to different characteristics for treatments, the results of which are presented in Table 3. In model 1, primary tumor size ≥5 cm (hazard ratio [HR]: 1.80; 95% CI: 1.28–2.53; *P* = .001) and serum AFP ≥200 (HR: 1.44; 95% CI: 1.14–1.81; *P* = .002) were significantly correlated with increased mortality. In addition, serum albumin ≥3.5 (HR: 0.65; 95% CI: 0.52–0.82; *P* < .001) was significantly correlated with decreased mortality. Furthermore, compared with sorafenib, resection (HR: 0.35; 95% CI: 0.18–0.58; *P* = .002) had a significantly stronger correlation with decreased mortality. Finally, BSC (HR: 1.82; 95% CI: 1.38–2.41; *P* < .001) was significantly associated with increased mortality. In model 2, primary tumor size ≥5 cm (HR: 1.76; 95% CI: 1.26–2.74; *P* = .001) and serum AFP ≥200 (HR: 1.44; 95% CI: 1.14–1.81; *P* = .002) were remarkably correlated with increased mortality. In addition, serum albumin ≥3.5 (HR: 0.68; 95% CI: 0.55–0.85; *P* = .001) was remarkably correlated with decreased mortality. Furthermore, compared with sorafenib alone, multiple-line treatments with sorafenib (HR: 0.70; 95% CI: 0.51–0.95; *P* = .023) and multiple-line treatments without sorafenib (HR: 0.49; 95% CI: 0.29–0.82; *P* = .007) had significantly stronger associations with decreased mortality. Finally, BSC (HR: 1.41; 95% CI: 1.01–1.96) was remarkably correlated with increased mortality. In model 3, primary tumor size ≥5 cm (HR: 1.79; 95% CI: 1.28–2.51; *P* = .002) and serum AFP ≥200 (HR: 1.40; 95% CI: 1.14–1.77; *P* = .001) were remarkably correlated with increased mortality. In addition, serum albumin ≥3.5 (HR: 0.68; 95% CI: 0.55–0.85; *P* = .001) was significantly associated with decreased mortality. Furthermore, compared with first-line treatment, second-line treatments (HR: 0.76; 95% CI: 0.58–0.98; *P* = .039) and third-line treatments (HR: 0.58; 95% CI: 0.37–0.91; *P* = .017) were significantly associated with decreased mortality. Finally, BSC (HR: 1.53; 95% CI: 1.15–2.03; *P* = .003) was significantly correlated with increased mortality.

**Table 2:**
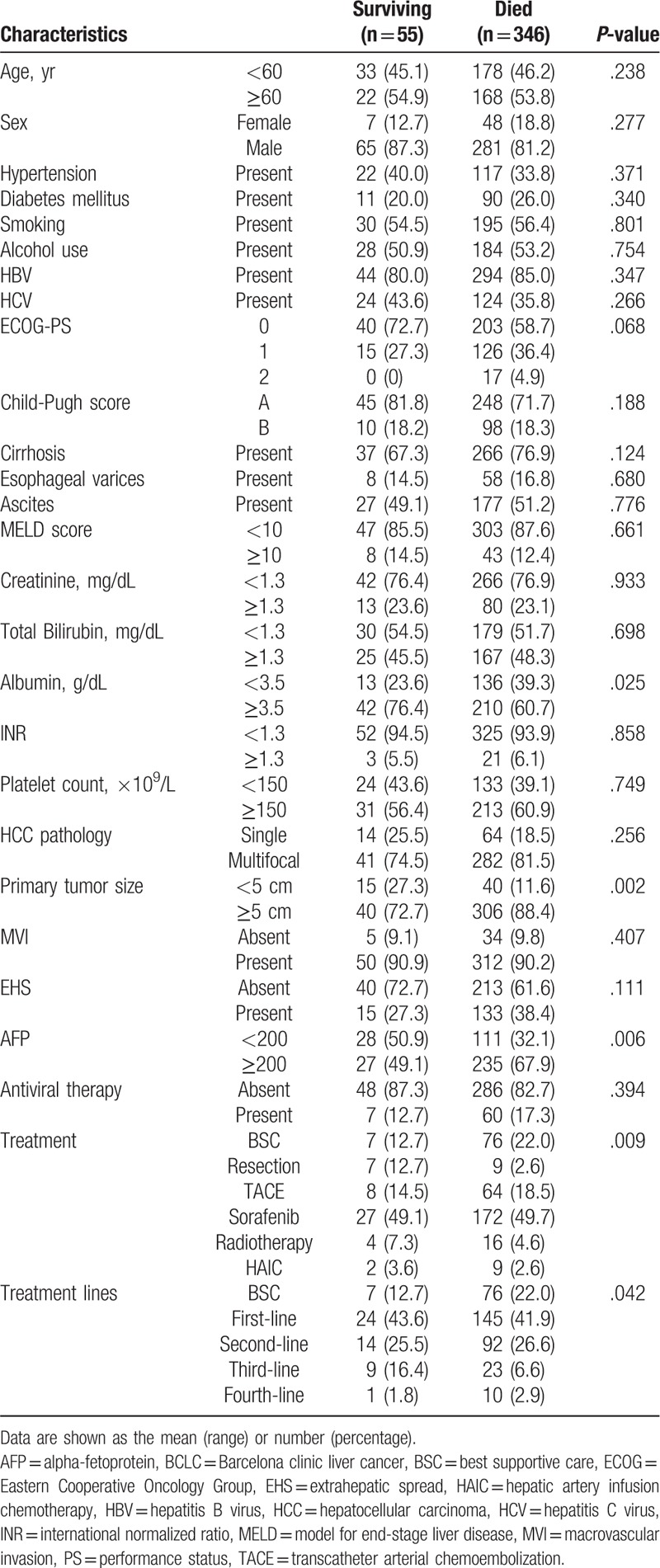
Univariate analysis of prognostic factors for mortality.

**Table 3:**
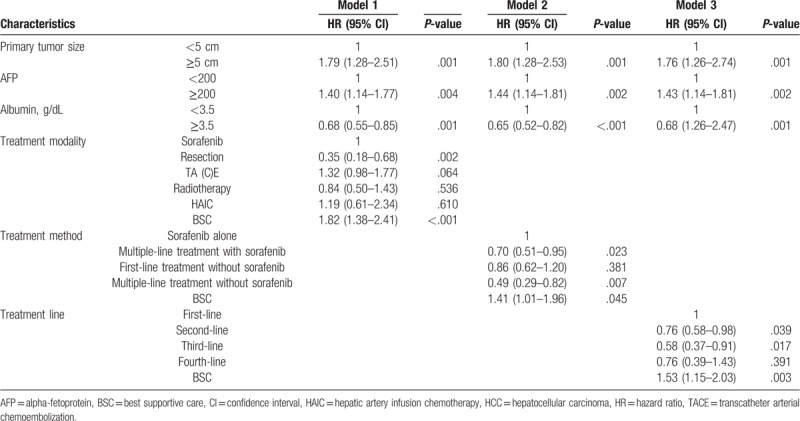
Multivariate Cox regression analysis of prognostic factors for mortality.

### 3.3 Treatment analysis

The treatment modality and lines are shown in Table 4. Of the treatment modalities, sorafenib (49.9%) was most frequently employed, followed by BSC (20.7%), transarterial therapies (18.0%), radiotherapy (6.7%), and resection (6.0). Of the treatment lines, first-line therapies (42.7%) were the most commonly used management, followed by second-line therapies (26.4%), BSC (20.7%), third-line therapies (8.0%), and fourth-line therapies (2.7%). In addition, sorafenib was the most common used management in the first-, second-, third-, and fourth-line treatment.

**Table 4:**
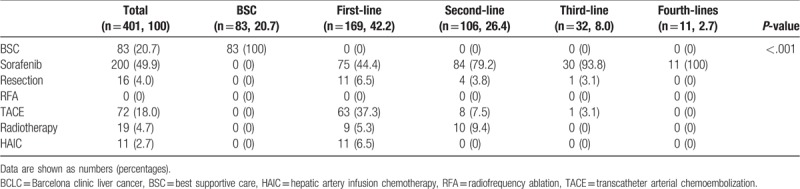
Treatment modality and lines.

### 3.4 OS of patients undergoing different treatments

**Figure 1:**
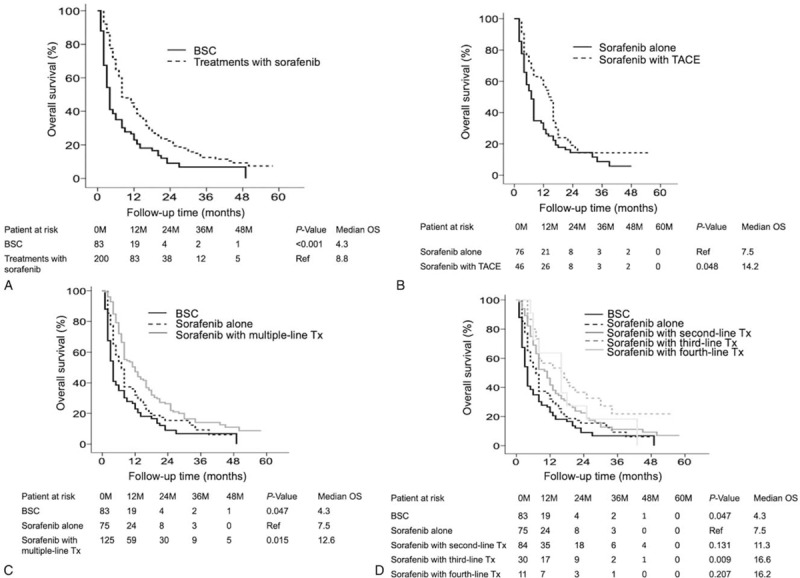
OS of patients with BCLC C stage HCC who underwent different sorafenib-based therapies, as assessed by Kaplan–Meier analysis. Treatment with sorafenib yielded a significantly better median OS than BSC (*P* < .001) (A). Sorafenib with concurrent TACE had a significantly better median OS than sorafenib alone (*P* = .048) (B). Sorafenib with multiple-line treatments resulted in a significantly better median OS than sorafenib alone (*P* < .015) (C). Sorafenib with third-line treatments yielded a significantly better median OS than sorafenib alone (*P* < .009) (D). BCLC = Barcelona clinic liver cancer, BSC = best supportive care, HCC = hepatocellular carcinoma, OS = overall survival, TACE = transarterial chemoembolization.

Assessment of sorafenib-based treatments indicated that sorafenib alone (8.8 months, 95% CI: 6.1–9.8, *P* < .001) yielded a remarkably longer median OS than BSC (4.3 months, 95% CI: 3.2–4.8) (Fig. 1A). In addition, sorafenib combined with TACE (14.2 months, 95% CI: 11–16, *P* = .048), multiple-line treatments with sorafenib (12.6 months, 95% CI: 8.0–15, *P* = .015), and third-line treatments with sorafenib (16.6 months, 95% CI: 3.9–28, *P* = .009) resulted in a remarkably longer median OS than sorafenib alone (7.5 months, 95% CI: 5.3–8.6) (Fig. 1B–D). The third-line treatments with sorafenib had the highest median OS (16.6 months), and among the third-line treatments, most of the treatments yielded a median OS concomitant with that of sorafenib, TACE, and radiotherapy.

**Figure 2:**
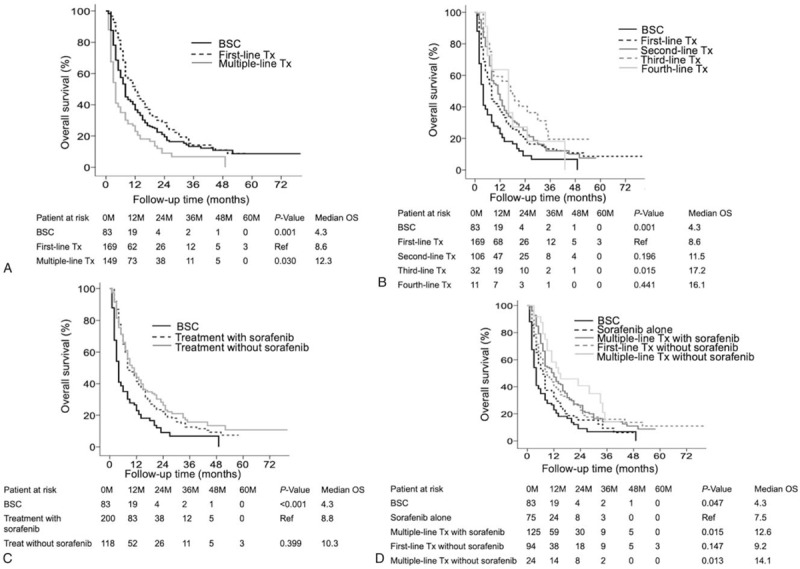
OS of patients with BCLC C stage HCC who underwent multiple-line therapies, as assessed by Kaplan–Meier analysis. Multiple-line treatments yielded a significantly better median OS than first-line treatment (*P* < .001) (A). Third-line treatments resulted in a significantly better median OS than first-line treatment (*P* = .015) (B). Treatments with or without sorafenib had a similar median OS (*P* < .001) (C). Multiple-line treatments with or without sorafenib yielded a significantly better median overall survival than sorafenib alone (*P* = .013) (D). BCLC = Barcelona clinic liver cancer, HCC = hepatocellular carcinoma, OS = overall survival.

Of the combination therapies, multiple-line treatments (12.3 months, 95% CI: 9.3–14.6) resulted in a remarkably longer median OS than either first-line treatment (8.6 months, 95% CI: 6.8–9.2, *P* = .03) or BSC (4.3 months, 95% CI: 3.2–4.8, *P* < .001) (Fig. 2A), and third-line treatments (17.2 months, 95% CI: 8.7–25) had a remarkably longer median OS than first-line treatment (8.6 months, 95% CI: 6.8–9.2, *P* = .015) (Fig. 2B). Therapies with (8.8 months, 95% CI: 6.1–9.8) and without sorafenib (10.3 months, 95% CI: 6.9–13) yielded a similar median OS, but both OS periods were longer than that of BSC (4.3 months, 95% CI: 3.2–4.8, *P* < .001) (Fig. 2C). Multiple-line treatments with or without sorafenib (12.6 months; 95% CI: 8.0–15, *P* = .015 and 14.1 months: 95% CI: 9.7–27, *P* = .013, respectively) yielded a remarkably longer median OS than sorafenib alone (7.5 months; 95% CI: 5.3–8.6) (Fig. 2D).

## 4 Discussion

Sorafenib is the only recommended treatment for patients with BCLC stage C HCC.^[7,14]^ Although sorafenib promotes OS in HCC patients with MVI and/or EHS, the clinical prognosis is still unsatisfactory.^[8]^ In addition, sorafenib in combination with TACE or locoregional therapies may increase OS in patients with advanced HCC.^[17,18,22,24]^ Our study demonstrated that primary tumor size ≥5 cm, serum AFP ≥200, and serum albumin <3.5 were significantly associated with increased mortality. In addition, compared with BSC, sorafenib alone prolonged the OS in patients with BCLC stage C HCC. Furthermore, compared with sorafenib alone, sorafenib with TACE, multiple-line treatments with sorafenib, third-line treatments with sorafenib, and multiple-line treatments without sorafenib significantly increased the median OS. Therefore, sorafenib with concurrent multiple-line therapies significantly improved the OS in patients with BCLC stage C.

The effectiveness of sorafenib in combination with TACE versus sorafenib alone in increasing the OS of patients with BCLC stage C disease is controversial. One study showed that compared with sorafenib alone, sorafenib with concurrent TACE improved the OS in patients with BCLC stage C HCC.^[21]^ However, some studies have shown that compared with sorafenib alone, sorafenib with concurrent TACE did not promote OS in BCLC stage C patients.^[23–25]^ Our study demonstrated that sorafenib with concurrent TACE significantly improved the median OS from 7.5 months to 14.2 months (*P* = .048) in BCLC stage C HCC. These results are consistent with those of a previous study.^[21]^ Sorafenib in combination with TACE seems to provide better OS if patients have well-preserved liver function and can tolerate these combination therapies. Moreover, many studies on BCLC stage C HCC have shown that compared with sorafenib alone, sorafenib in combination with TACE promotes the tumor response rate, progression-free survival, and time to progression.^[23–27]^

A randomized controlled trial in a Western country demonstrated that in HCC patients with portal vein thrombosis, sorafenib combined with RFA results in better OS than sorafenib alone.^[17]^ Our study showed that compared with sorafenib alone, sorafenib in combination with multiple-line therapies, including surgical resection, TACE, RFA, radiotherapy or HAIC, resulted in a significantly higher median OS. Furthermore, third-line treatments with sorafenib had the highest median OS (16.6 months). This finding implies that combining sorafenib with locoregional therapy promotes the tumor response and increases OS. The possible reason is that locoregional therapies such as TACE block the feeding vessel, which results in tumor hypoxia. Sorafenib suppresses tumor cell neoangiogenesis and delays tumor progression in HCC patients. In addition, local treatments, including surgical resection, TACE, or RFA, can induce the overproduction of vascular endothelial growth factor (VEGF), which may aggravate the tumor or metastases. Hence, sorafenib may enhance the treatment results by decreasing VEGF overexpression when sequentially administered after local therapies.

Our study showed that multiple-line therapies without sorafenib yielded a better OS (14.2 months) than other therapies. In some cases, patients were not administered sorafenib because of patient intolerance or refusal to take the drug. Several studies have demonstrated that surgical resection significantly favors survival in HCC patients with major vascular invasion, including those with invasion into the portal and hepatic veins.^[28–30]^ RFA and TACE can increase the partial response of tumors, and HAIC should be considered a treatment option for patients with advanced HCC.^[31]^ Radiotherapy may decrease tumor progression and offer survival benefits in HCC patients with portal vein thrombosis.^[32,33]^ The combination of RFA, TACE, HAIC, and radiotherapy can increase the tumor response and delay disease progression. In some cases, these therapies can serve as a bridge to surgical resection. The combination of these therapies should be considered as preoperative treatment modalities and should improve OS in advanced HCC.

Our study has several limitations. First, there were significant gender differences in this investigation, but we will enroll more female subjects and reduce the bias. Second, patients may have accepted multimodal sequential therapies and different treatment modalities, which would affect survival. The optimal use and timing of these combination therapies need to be further studied. Third, we did not mention the duration or side effects of sorafenib due to the retrospective nature of this study.

## 5 Conclusion

The primary tumor size, serum AFP, and serum albumin were significantly associated with mortality. In addition, sorafenib with TACE, multiple-line treatments with sorafenib, third-line treatments with sorafenib, and multiple-line treatments without sorafenib resulted in a significantly better median OS than sorafenib alone. Sorafenib with concurrent multiple-line therapies significantly improved survival in advanced HCC and was demonstrated to have a manageable efficacy in advanced HCC patients with well-preserved liver function. In the future, sorafenib in combination with locoregional therapies will provide important information for immunotherapy combinations with locoregional therapies in advanced HCC.

## Author contributions

**Conceptualization:** Chih-Wen Lin.

**Data curation:** Pojen Hsiao, Yaw-Sen Chen, Chia-Chang Hsu.

**Formal analysis:** Pojen Hsiao, Kun-Chou Hsieh, Yaw-Sen Chen, Chia-Chang Hsu.

**Investigation:** Gin-Ho Lo, Yu-Chan Li, Chih-Wen Lin.

**Methodology:** Gin-Ho Lo, Yu-Chan Li, Pei-Min Hsieh, Kun-Chou Hsieh, Hung-Yu Lin.

**Project administration:** Chih-Wen Lin.

**Resources:** Chih-Wen Lin.

**Software:** Tsung-Chin Wu, Jen-Hao Yeh.

**Supervision:** Chih-Wen Lin.

**Validation:** Tsung-Chin Wu, Jen-Hao Yeh.

**Visualization:** Gin-Ho Lo, Tsung-Chin Wu, Jen-Hao Yeh.

**Writing – original draft:** Pojen Hsiao, Yaw-Sen Chen, Chih-Wen Lin.

**Writing – review and editing:** Pojen Hsiao, Kun-Chou Hsieh, Yaw-Sen Chen, Chih-Wen Lin.

Chih-Wen Lin orcid: 0000-0003-2344-5056.
